# Modelling the impact of improving screening and treatment of chronic hepatitis C virus infection on future hepatocellular carcinoma rates and liver-related mortality

**DOI:** 10.1186/1471-230X-14-137

**Published:** 2014-08-07

**Authors:** Matthew E Cramp, William M Rosenberg, Steven D Ryder, Sarah Blach, Julie Parkes

**Affiliations:** 1 Hepatology Research Group, Peninsula Medical School and Hepatology Department, South West Liver Unit, Derriford Hospital, Plymouth, UK; 2 Institute for Liver and Digestive Health, Division of Medicine, University College London, Royal Free Campus, Rowland Hill Street, London NW3 2PF, UK; 3 NIHR Biomedical Research Unit in Gastrointestinal and Liver Diseases, Nottingham University Hospitals NHS Trust and The University of Nottingham, Nottingham NG7 2UH, UK; 4 Center for Disease Analysis, Louisville, CO 80027, USA; 5 Public Health Sciences and Medical Statistics, Faculty of Medicine, University of Southampton, Southampton SO16 6YD, UK

**Keywords:** Hepatitis C virus, Hepatocellular carcinoma, Cirrhosis, Decompensated cirrhosis

## Abstract

**Background:**

The societal, clinical and economic burden imposed by the complications of chronic hepatitis C virus (HCV) infection - including cirrhosis and hepatocellular carcinoma (HCC) - is expected to increase over the coming decades. However, new therapies may improve sustained virological response (SVR) rates and shorten treatment duration. This study aimed to estimate the future burden of HCV-related disease in England if current management strategies remain the same and the impact of increasing diagnosis and treatment of HCV as new therapies become available.

**Methods:**

A previously published model was adapted for England using published literature and government reports, and validated through an iterative process of three meetings of HCV experts. The impact of increasing diagnosis and treatment of HCV as new therapies become available was modelled and compared to the base-case scenario of continuing current management strategies. To assess the ‘best case’ clinical benefit of new therapies, the number of patients treated was increased by a total of 115% by 2018.

**Results:**

In the base-case scenario, total viraemic (HCV RNA-positive) cases of HCV in England will decrease from 144,000 in 2013 to 76,300 in 2030. However, due to the slow progression of chronic HCV, the number of individuals with cirrhosis, decompensated cirrhosis and HCC will continue to increase over this period. The model suggests that the ‘best case’ substantially reduces HCV-related hepatic disease and HCV-related liver mortality by 2020 compared to the base-case scenario. The number of HCV-related HCC cases would decrease 50% by 2020 and the number progressing from infection to decompensated cirrhosis would decline by 65%. Therefore, compared to projections of current practices, increasing treatment numbers by 115% by 2018 would reduce HCV-related mortality by 50% by 2020.

**Conclusions:**

This analysis suggests that with current treatment practices the number of patients developing HCV-related cirrhosis, decompensated cirrhosis and HCC will increase substantially, with HCV-related liver deaths likely to double by 2030. However, increasing diagnosis and treatment rates could optimise the reduction in the burden of disease produced by the new therapies, potentially halving HCV-related liver mortality and HCV-related HCC by 2020.

## Background

Chronic Hepatitis C Virus (HCV) infection can induce hepatic inflammation, which potentially results in progressive fibrosis leading to cirrhosis [1], and hepatocellular carcinoma (HCC) [2]. These complications typically develop slowly: untreated, approximately 10% of people develop cirrhosis within 10 years when infected as young adults [3], increasing to about 33% within 20 years [1].

HCV-related complications are increasingly common. For example, hospital admissions in England from HCV-related end-stage liver disease (ESLD) and HCC rose approximately four-fold from 574 in 1998 to 2266 in 2012 [4]. Deaths from HCV-related ESLD and HCC increased from 89 to 326 between 1996 and 2012 [4]. The number of people living with HCV-related decompensated cirrhosis or HCC in England is projected to increase about seven-fold from 590 in 1995 to 4210 in 2020 [4]. Therefore, the societal, clinical and economic burdens imposed by untreated HCV are expected to continue to rise in coming years as more patients progress to advanced liver disease.

Antiviral treatment of chronic HCV aims to produce a sustained virological response (SVR), defined as undetectable levels of HCV RNA 12 or 24 weeks after treatment. SVR is associated with reduced all-cause [5] and liver-related mortality [5,6], and HCC rates [7]. Dual therapy with pegylated interferon (peg-IFN) and ribavirin produces SVR in approximately 80% of genotype 2 and 3 infections and 40-50% for genotype 1 [8,9]. Adding either boceprevir or telaprevir - directly acting antivirals (DAAs) – to dual therapy improves the overall SVR for genotype 1 by at least 20-25% in treatment-naïve patients [9].

Against this background, new DAAs raise the prospect of improved SVR rates, fewer adverse events and shorter treatment regimens [10-12]. Indeed, an peg-IFN-free, pan-genotypic and all-oral regimen appears to be a realistic possibility [12]. However, new therapies may not ease the burden of disease unless more people with HCV are detected and treated. Currently, several barriers hinder optimal care, including: low diagnosis rates; the asymptomatic nature of chronic HCV before complications emerge; social stigma; failure to refer to specialist services; comorbidities; patient concerns about side effects; and suboptimal adherence to treatment [13-19]. Treatment rates among certain hard-to-reach patients, such as injecting drug users (IDUs) are particularly poor [20].

This analysis projects the burden of disease in England associated with current HCV management strategies until 2030 and models the impact of improved diagnosis and treatment as new therapies become available.

## Methods

The model, constructed by the Center for Disease Analysis (Colorado, USA), has been described in more detail elsewhere [21]. The model (Microsoft Excel) tracks chronic HCV progression between 1950 and 2030 by five-year age and gender cohorts, and by liver-disease stage. Newly infected patients can enter the model at any year, progress through the disease stages based on published transition probabilities [22], and exit the model on: spontaneous clearance of HCV; achieving SVR; or death (all-cause or HCV-related). Historical inputs collected from published literature and reports published by the English government were used to populate and calibrate the model. For example, the model has been validated using data from the Office for National Statistics (ONS) and Public Health England (PHE) [4,23,24]. Between 1998 and 2012 the model correlates closely with reported hospital admission data for ESLD and HCC [4], but provides a more conservative estimate of each . The model does not distinguish previously treated patients and naïve patients. However, average SVR rates used in the model encompass previously treated patients, naïve patients and patients treated at advanced stages of disease (F2-F4).

The base-case scenario modelled continuing the current management strategy on HCV-related disease, in particular HCC. The base case was compared against a model of increasing diagnosis and treatment rates as new therapies become available.

### English population data

English population estimates were calculated as a percent of the total UK population, obtained as 5-year age and sex cohorts based on the United Nations (UN) population database [25]. The UN database combines reliable local data sources and contained estimates for 1950 to 2100 in a format consistent with the model (i.e. annual 5-year age and gender cohorts). A limitation of this approach was the need to estimate the English population from the total UK population figures. The percentage of the UK population residing in England in 1991, 2001 and 2011, was obtained from the ONS and was used to estimate the English population [26].

### Characteristics of the HCV population

**Figure 1:**
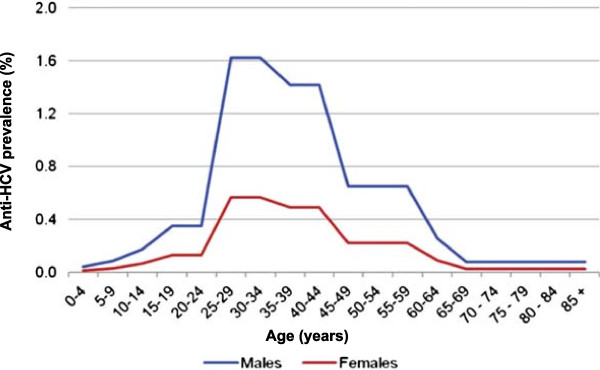
**The age and gender distribution of HCV in England in 2005 **[27]**.**

PHE, formerly the Health Protection Agency (HPA), uses a Bayesian model to produce annual estimates of HCV prevalence in England and Wales [27]. A published evidence synthesis estimated the excess risk among ethnic minorities to generate a more robust calculation of HCV prevalence, based on the presence of antibodies, in England: 0.54% (95% confidence interval [CI]: 0.4%-0.75%) among the English population over 15 years of age in 2005 [27]. This is equivalent to 0.4% (95% CI: 0.3%-0.6%) of the total population, including people less than 15 years of age, in England. Figure 1 shows the HCV prevalence in England in 2005 stratified by age and gender.

The present model includes only viraemic cases (defined as patients who test positive for HCV RNA). Antibodies indicate previous HCV exposure, not necessarily current infection. Therefore, limiting the model to individuals positive for HCV RNA ensures that the model includes only current infections. A rate of 68.7% was used [28] to calculate the proportion of people with antibodies to HCV that are also RNA positive. This corresponded to 151,600 (95% CI: 105,100–196,500) viraemic cases in 2005 equivalent to a prevalence of 0.3% (95% CI: 0.2%-0.4%) of the English population. HCV genotypes 1 and 3 account for 45% and 46% of infections in England respectively, according to data collected by 18 sentinel centres between 2007 and 2010 [28]. Table 1 shows the full genotype distribution.

**Table 1:** **Distribution of HCV genotypes 1–4 in England by percentage **[28]

**Genotypes**	**1a**	**1 other**	**2**	**3a**	**3 other**	**Other**
Distribution	23.0%	21.7%	5.7%	33.4%	12.5%	3.9%

The annual number of new cases in England peaked in 1990 due to infections associated with blood products, then decreased after the introduction of blood screening [29]. The model also included a second peak in 2000 to reflect self-reported needle sharing between 1991 and 2011 [30]. IDU accounted for 90% of laboratory confirmed HCV cases between 1996 and 2012 [4]. The remaining risk factors reported by diagnostic laboratories in England during this period were: blood transfusion or receiving a blood product (2% of laboratory confirmed HCV cases); sexual exposure (2%); renal failure (0.4%); vertical transmission from mother to child; household (0.2%); occupational (0.2%); and other (4.9%) [4].

### HCV-related morbidity and mortality

The model accounts for mortality in the general HCV population and a high-risk subpopulation. Mortality in the high-risk population may be higher than in the background population because of causes unrelated to HCV (e.g. overdoses and accidents). Active IDUs are a high-risk population and, therefore, an increased mortality rate was applied to ensure that the model does not overestimate the burden of disease. Background mortality was estimated using a database hosted by the Max Planck Institute for Demographic Research [31]. We assumed that the peak IDU occurs between the ages of 15 and 44 years. Therefore, increased mortality among active IDUs was estimated for individuals between 15 and 44 years of age [32-37].

### Diagnosis inputs for the base-case scenario

HCV notification reports from 1992–2010 (n = 8,147) [38] were adjusted to account for mortality and for patients who were cured (i.e. no detectable HCV RNA after antiviral treatment), so that only individuals with current chronic infection remained. The number of cured patients was calculated by multiplying the number of treated patients each year by the average SVR. Based on these figures, an estimated 67,200 individuals had been identified living with an antibody-based HCV diagnosis in 2010. Adjusting this value using a viraemic rate of 68.7%, an estimated 46,200 individuals had been identified with RNA-confirmed HCV in 2010. Each year, an estimated 5600 individuals are newly diagnosed with chronic HCV. SVR inputs for the base-case were set at 70% for genotypes 1–3 and 48% for genotype 4 in 2013 (Table 2). The average SVR for genotype 1 took into consideration treatment with protease inhibitors (ie boceprevir and telaprevir).

**Table 2:** Inputs for the scenario testing the impact of increasing diagnosis and treatment of HCV

**Inputs**	**2013 (Base)**	**2014**	**2016**	**2018 and after**
SVR G1	70%	80%	95%	95%
SVR G2	70%	85%	95%	95%
SVR G3	70%	70%	80%	90%
SVR G4	48%	80%	95%	95%
Age	15 - 64	15 - 64	15 - 69	15 - 74
Eligibility	60%	60% - 80%	80%	95%
Treated	5430	8150 [F2]	10190 [F2]	11710 [F1]
Diagnosed	5600	8400	13430	13430
Age	15 - 64	15 - 69	15 - 74	15 - 74

### Treatment numbers for the base-case scenario and future increases in SVR

The model estimates that 5,430 HCV patients were treated in England during 2010. This was based on the number of standard units of peg-IFN sold between 2006 and 2011, with an estimated 26,670 patients treated in this period (IMS Health Incorporated; Danbury, Connecticut). This estimate is in line with those of PHE, which estimates that a total of 27,500 patients were treated between 2006 and 2011 [4]. The English genotype distribution was used to estimate the average number of weeks of treatment and, therefore, the number of units of peg-IFN used per patient, assuming 80% adherence. The base-case scenario assumed that the diagnosis rate, treatment rate and SVR remained constant from 2013 to 2030.

Once the base case was developed, the model was used to analyse alternative inputs for the number of patients treated, eligibility, age-related treatment restrictions, SVR by genotype (G1, G2, G3, G4), and the total number of newly diagnosed HCV cases at different times. This modelled the impact of increasing rates of diagnosis and treatment of HCV as new DAAs become available.

The model examined the impact of future therapies becoming available in four waves:

•In 2014, new DAAs (e.g. an NS5B inhibitor) become available that are combined with peg-IFN and ribavirin for genotypes 1, 3 and 4. The model for genotype 2 assumed the new DAA is combined with ribavirin only.

•In 2016, further new DAAs are combined with ribavirin for genotypes 1–4.

•In 2018, ribavirin-free all-oral combinations of DAAs will be available.

•In 2020, third-generation DAA combinations will become available.

The model assumed that uptake of new therapies was immediate. To reflect the increased efficacy of new therapies, the average SVR was modified for each wave of new therapies (Table 2).

### Increasing diagnosis and treatment of HCV

The model estimated the impact of increasing the number of patients that are treated with new therapies on HCV-related morbidity and mortality. To estimate the ‘best case’ of producing an optimal impact with new therapies on the burden of disease, increases in the number of treated patients were required. For the purposes of this analysis, the number of patients treated was modelled to increase from 5,430 in 2013 to 8,150 in 2014. The number of patients treated then increased to 10,190 in 2016, and 11,710 in 2018 (Table 2). This increase in treatment was selected to accomplish a substantial impact of new therapies on the burden of HCV infection. The increase in treated patients was initially modelled in those with F2 or higher fibrosis, before the treated cohort included F1 or higher from 2018 to provide sufficient numbers of patients to be treated. In the model, eligibility refers to the number of patients without contraindications who are willing to accept treatment. New therapies were assumed to increase eligibility for treatment, increasing from 60% in 2013 to 95% in 2018. In addition, the upper age-limit for treatment increased from 69 years old to 74 years old in 2016 (Table 2). To allow for increases in treatment, the model required that the number of patients newly diagnosed increased from 5,600 in 2013 to 8,400 in 2014 and to 13,430 in 2016 and beyond (Table 2).

### Sensitivity analyses

Sensitivity analyses and Monte Carlo simulations were performed using Crystal Ball, an Excel add-in by Oracle, to quantify the impact of uncertainties on modelled outcomes. Under Monte Carlo simulation, uncertain variables are represented as probability distributions and model outputs are recalculated 1,000 times to estimate a range of possible outcomes. Each recalculation uses a new randomly selected set of values from the input probability distributions and the likelihood of each outcome is recorded to generate 95% uncertainty intervals (95% UI). Beta-PERT distributions were used for all uncertain variables. Additionally, the impact of variations in the assumptions made in the ‘best case’ scenario (including the number of treated and newly diagnosed patients, and the segment of the population treated) on HCC cases and the number of liver-related deaths by 2020 was measured.

## Results

### Base case

**Figure 2:**
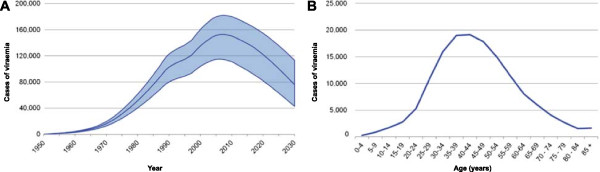
**Outputs from the base case.** The number of viraemic cases of HCV in England from 1950 to 2030 **(A)** and the age distribution of viraemic cases in England in 2013 **(B)**.

The model estimated that there were 144,000 (95% UI: 103,000-174,000) viraemic cases of HCV in England in 2013 (Figure 2A). The age distribution in 2013 is shown in Figure 2B and estimates for treatment and diagnosis in 2013 are shown in Table 3.

**Table 3:** Summary of the historical inputs and estimates for the HCV population in the base case

**Inputs**	**Historical (confidence interval)**	**Year**	**2013 (uncertainty interval)**
HCV infected cases	220720 (153000–286000)	2005	
Antibody HCV prevalence	0.4% (0.3% - 0.6%)		
Total viraemic cases	151640 (105110–196480)	2005	144000 (103000–174000)
Viraemic prevalence	0.3% (0.2% - 0.4%)		0.3%
Viraemic rate	68.7%		68.7%
HCV diagnosed (Viraemic)	46200	2010	49730
Viraemic diagnosis rate	30.4%		34.5%
Annual newly diagnosed	5600	2010	5600
New infections			3980
New infection rate (per 100 K)			7
Treated			
Number treated	5430	2011	5430
Annual treatment rate	3.6%		3.8%

Peak viraemic prevalence of chronic HCV infection was reached in 2007 with 153,000 infected individuals. Since then, the model suggested a decline in overall prevalence reaching an estimated 76,300 in 2030 (Table 4). Due to the lag between infection and the onset of HCV-related hepatic complications, the number of cases of compensated cirrhosis is projected to peak in 2029 at 14,800, increasing from 9,500 in 2013 (Table 4). The population with decompensated cirrhosis is estimated to increase from 860 in 2013 and peak in 2029 at 1400 cases. The number of individuals with HCV-related HCC is projected to increase from 410 in 2013 to 880 in 2030 (Table 4). Unless current practices of care in England change, the model forecasts that liver-related mortality will increase by 90% to 740 by 2030 (Table 4).

**Table 4:** Outcomes of the base-case scenario at 2013 and 2030

**Base-case outcomes (year)**	**Total HCV cases**	**HCV-related fibrosis**	**HCV-related cirrhosis**	**HCV-related decompensated cirrhosis**	**HCV-related HCC**	**HCV-related liver related deaths**
2013	144000	133000	9500	860	410	390
	(103000–174000)	(95200–162000)	(4000–16600)	(350 – 1630)	(200–770)	(170–700)
2030	76300	59500	13700	1280	880	740
	(35300 – 106000)	(26500 – 89400)	(6370 – 20900)	(550 – 2150)	(430 – 1510)	(350 – 1140)

### The impact in 2020 of increasing diagnosis and treatment of HCV

**Figure 3:**
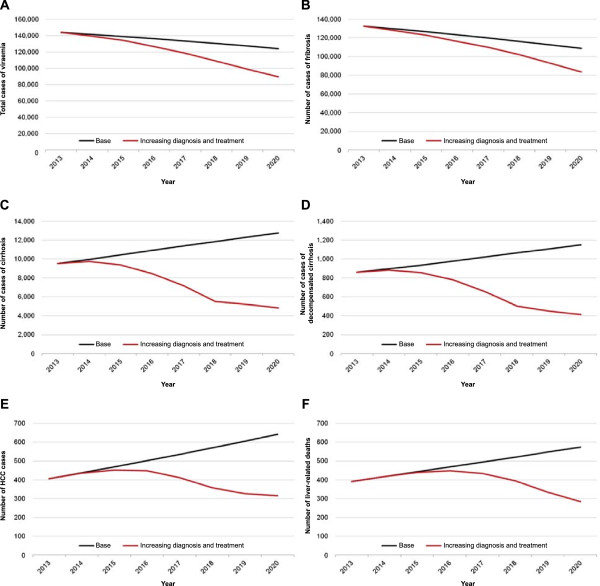
**HCV-related morbidity and mortality from 2013 to 2020.** The impact of increasing diagnosis and treatment of HCV compared to the base-case scenario on the total number of infected cases **(A)**, the number of patients with HCV-related fibrosis **(B)**, HCV-related cirrhosis **(C)**, HCV-infected decompensated cirrhosis **(D)**, HCV-related HCC **(E)** and the number of liver-related deaths caused by HCV **(F)** is illustrated. Sensitivity analyses - the effect of different treatment options on HCV-related HCC and mortality.

Table 5 compares the impact of increasing diagnosis and treatment of HCV as new therapies become available compared to continuing with the current strategy. In the latter case, the model predicts a total of 122,000 viraemic cases of HCV in 2020. Improving diagnostic and treatment rates by a total of 140% and 115% in 2018 respectively, would reduce the number of cases by 30% to 89,400 (Figure 3A).

**Table 5:** Summary of the impact of each treatment scenario at 2020

**2020 outcomes**	**Total HCV cases**	**HCV-related fibrosis**	**HCV-related cirrhosis**	**HCV-related decompensated cirrhosis**	**HCV-related HCC**	**HCV-related liver related deaths**
Base case	122000	107000	12600	1140	640	570
	(80300 – 153000)	(69900 – 138000)	(5560 – 20600)	(470 – 2050)	(320 – 1180)	(260–970)
Increasing diagnosis and treatment	89400	83500	4850	410	310	280
	(52300 – 122000)	(48900 – 116000)	(2050 – 10400)	(160 – 980)	(140 – 700)	(120 – 600)

Similarly, by increasing diagnosis and treatment, the number of HCV-related fibrosis cases in 2020 would decrease by 20% compared to the base case from 107,000 to 83,500 (Figure 3B). The number of patients with HCV-related cirrhosis and decompensated cirrhosis in 2020 would also decrease compared to base case, declining from 12,600 to 4,850 cases (60% reduction) and from 1,140 to 410 cases (65% reduction) respectively (Figure 3C and D). Meanwhile, the number of HCV-related HCC cases would decline from 640 to 310, a 50% reduction (Figure 3E). Finally, liver-related deaths caused by HCV would decrease by 50% from 570 to 280 cases in 2020 (Figure 3F).

### Sensitivity analyses

Monte Carlo analysis identified the range around the prevalence estimate as the largest driver of uncertainty in the model, accounting for 95% of explained variability. The results of the sensitivity analyses are shown in Table 6. Changes in the number of treated patients (from 12,000 annually to 3,000 annually) had the largest impact on HCC cases and HCV liver-related deaths in 2020. The disease state of treated patients (treating all ≥ F1 patients) and the age of the treated patients considered eligible for treatment (limiting to patients ≤69 years) showed the next largest impact on HCC cases and HCV liver-related deaths in 2020.

**Table 6:** Sensitivity analyses - the effect of different treatment options on HCV-related morbidity and mortality

	**Inputs**	**Number of HCV-related HCCs in 2020 (percentage change from scenario in Table **2**)**	**Liver-related deaths caused by HCV in 2020 (percentage change from scenario in Table **2**)**
Scenario: increasing diagnosis and treatment (Table 2)	--	310	280
Change segment treated	Allow treatment of F1	400 (30%)	390 (40%)
	Restrict treatment to ≥ F2	260 (−15%)	270 (−5%)
Change age of treated	Allow treatment of 79+	260 (−15%)	260 (−5%)
	Restrict treatment to 69	400 (30%)	330 (20%)
Change in the number treated (current- 12,000 in 2018)	Treat 3,000 (75% fewer)	590 (90%)	540 (95%)
	Treat 6,000 (50% fewer)	470 (50%)	430 (55%)
	Treat 9,000 (25% fewer)	360 (15%)	330 (20%)
	Treat 15,000 (25% more)	300 (−5%)	280 (0%)
	Treat 18,000 (50% more)	290 (−5%)	280 (0%)
	Treat 21,000 (75% more)	280 (−10%)	270 (−5%)
Change in the number diagnosed (Dx) (current- 13,000 in 2016)*	Dx 3,000 (75% fewer)	410 (30%)	370 (30%)
	Dx 7,000 (50% fewer)	350 (15%)	320 (15%)
	Dx 10,000 (25% fewer)	330 (5%)	200 (5%)

*Increasing the diagnosis rate above 13,000 had no effect under the current treatment levels and so has not been included.

## Discussion

This analysis predicts that if current HCV management in England remains the same between 2013 and 2030, the number of patients who will develop cirrhosis due to HCV will increase from 9,500 to 13,700 and cases of HCV-related decompensated cirrhosis will rise from 860 to 1,280. On the other hand, the model suggests that treating more patients with HCV will reduce the number of cases of HCV-related advanced hepatic diseases – including deaths from HCC and liver-related mortality – in England.

Improved referral and new treatments may increase the number of patients who can be treated. Optimising the clinical impact will require increases in treatment numbers of 50% across genotypes 1–4, from 5,430 to 8,150 in 2014 and by an additional 25% in 2016 to 10,190. A final increase of 15% in 2018 would bring the total to 11,710, a value that needs to be sustained until 2020 to optimise the clinical impact. The study planning and review meetings reached a consensus that the initial increase in treatment will enrol patients with an F2 or higher stage of fibrosis before being expanded to include F1 in 2018.

PHE modelled the impact on liver disease and HCC of increasing treatment of HCV with peg-IFN and ribavirin. Based on increasing the number of patients treated by 100%, PHE estimated that an additional 190 cases of ESLD and HCC could be prevented over ten years. PHE also calculated that from 2006 to 2011 27,500 HCV patients in England were probably treated based on purchasing and prescribing data for peg-IFN, equivalent to approximately 20% of the prevalent pool [4]. We calculated that 5430 HCV patients were treated annually in England using peg-IFN, which is in line with the PHE estimates [4]. However, the PHE assumed that peg-IFN and ribavirin would be the future standard of care, while this analysis also measured the use of the protease inhibitors boceprevir and telaprevir in genotype 1. The PHE report noted that if new therapies are easier for patients to tolerate then increasing treatment rates will become more feasible [4]. Therefore, our analysis probably offers a more realistic indication of potential health gains than the PHE estimates.

Reducing premature mortality from liver disease and cancer are important outcome measures for the National Health Service (NHS) [39]. The analysis shows that increasing treatment numbers by 50% in 2014 and by a further 44% in 2018 is likely to reduce liver-related mortality among HCV-infected patients by 50% in 2020 compared to the projections of current practices. This scenario depends on increases in SVR and diagnosis, and assumes that patients with advanced fibrosis (≥F2) are treated before those with less advanced fibrosis. The number of HCV-related HCC cases would also decrease by 50% in 2020. In addition, in 2020 the number of patients progressing to decompensated cirrhosis would decrease by 65%. The model predicts that the total number of HCV RNA-positive patients will decline by 30% compared to the projected impact of current treatment strategies.

Sensitivity analyses examined the impact of a range of widely variable assumptions on HCV-related morbidity and mortality compared to the scenario that modelled increased diagnosis and treatment of HCV. For example, changing the number of diagnosed patients to 75% fewer than that modelled in the scenario (to 3,000 from 13,000 cases) increased the number of HCV-related HCCs and liver-related deaths by 30%. Additionally, changing the number of treated patients to 75% fewer than that modelled in the scenario to 75% above that modelled (3,000 to 21,000 cases) altered the number of HCV-related HCCs from 90% above to 10% below the modelled scenario. The number of liver-related deaths caused by HCV changed from 95% above to 5% below respectively. Sensitivity analysis showed that increasing treatment numbers to 21,000 patients annually had a small impact on the number of HCC cases and liver-related mortality in 2020 compared to the reductions already achieved in the ‘best case’ scenario. The number of treated patients selected in the ‘best case’ scenario (11,710 by 2018) was chosen to minimise the impact of morbidity and mortality. However, further increases in treatment numbers would have a large impact on the total number of infections in England if eligibility is increased to patients with F0-F1 stage of fibrosis.

This analysis focused on chronically infected individuals. Once a patient achieved SVR they were removed from the infected cohort. Although studies have shown that patients with SVR retain some risk of HCC, decompensation and liver-related death, the rate of progression is substantially lower than in patients with current infection [40]. Additionally, because patients achieving SVR are not tracked, all re-infections in the model are handled as naïve cases. Combined, these two limitations suggest that the model may overestimate the impact that SVR has on HCV liver-related morbidity and mortality. However, any overestimate is likely to be limited. Firstly, the modelled scenario aims to cure patients before they progress to advanced disease, thus lowering the risk of disease progression after cure. Secondly, the risk of HCV re-infection is estimated to be small even in IDUs [41].

Furthermore, the analysis did not model healthcare or treatment costs. Mean healthcare costs associated with HCV increase as the disease progresses, underscoring the importance of treating patients before they progress to advanced disease [42]. Future analysis including costs incurred by any service redesign to enable greater testing and treatment, and indirect costs (e.g. loss of productivity) or intangible costs (e.g. distress or pain) would be beneficial. The analysis also assumes that there is immediate uptake of new therapies once they become available. In reality, the uptake of treatment can be delayed as new practice guidelines are developed and healthcare professionals become used to new protocols and gain clinical experience with new agents.

An additional limitation is that the increased mortality rate was applied only to those estimated to be actively injecting drugs between the ages of 15 and 44 years. Deaths among active IDUs are usually due to non-HCV-related factors, such as suicides, overdoses and accidents [34,43]. However, mortality due to IDU may persist after this age due to, for example, HIV, hepatitis B, other addictions and risk-taking behaviour. In addition, drug use may persist after 44 years of age. However, this limitation means that the model is likely to underestimate the benefits of increasing HCV-treatment rates, for example, ex-IDUs with HCV may not be known to services.

Maximising the potential reductions in HCV-related mortality and morbidity associated with new treatments and service structures means overcoming barriers to care [14,16]. For example, we estimated that 49,730 viraemic individuals were living with a diagnosis of HCV in 2013, equating to a viraemic diagnostic rate of 34.5%. Therefore, most infected individuals are unaware of their status. For example, in a sample of HCV-infected IDUs approximately half were unaware of their status [4]. The diagnostic rate is less than the estimated rate in Scotland, where approximately 52% of those with chronic HCV infection were thought to have been diagnosed [4]. The limited publicity of HCV testing and minimal screening for HCV in high-risk groups in England may account for the difference. For example, a commitment to deliver ‘opt-out’ testing for blood-borne viruses in prisons in England was agreed only in April 2014 [44].

## Conclusions

In conclusion, this analysis demonstrated that if current HCV treatment practices in England continue, the number of patients with HCV-related cirrhosis, decompensated cirrhosis and HCC can be expected to rise over the coming years. However, under a best-case scenario improved treatment and diagnosis offer the opportunity to halve HCV-related liver mortality and HCV-related HCC by 2020. Realising these benefits in England will probably require service redesign to remove current barriers to care.

## Competing interests

This project received financial support from Gilead Sciences Ltd., who had no input into study design or data selection. MEC has sat on advisory boards and participated in clinical trials for Gilead Sciences Ltd, Janssen-Cilag Ltd, Boehringer Ingelheim Ltd UK, Bristol-Myers Squibb Company, Merck & Co., Inc and F. Hoffmann-La Roche Ltd. SDR has been a member of advisory boards for Gilead Sciences Ltd, Merck Sharp & Dohme Corp., Boehringer Ingelheim Ltd UK, and F. Hoffmann-La Roche Ltd. WMR has served on advisory boards for Gilead Sciences Ltd, Janssen-Cilag Ltd, Bristol-Myers Squibb Company, Merck Sharp & Dohme Corp., Hoffmann-La Roche Ltd and GlaxoSmithKline. SB is an employee for the Center for Disease Analysis.

## Authors’ contribution

MEC took part in the study planning and review meetings, helping to validate and select the inputs used to populate the model. MEC contributed to the planning, review and revision of the manuscript. WMR took part in the study planning and review meetings, helping to validate and select the inputs used to populate the model. WMR contributed to the planning, review and revision of the manuscript. SDR took part in the study planning and review meetings, helping to validate and select the inputs used to populate the model. SDR contributed to the planning, review and revision of the manuscript. SB carried out the modelling involved in this study. SB drafted the methods and results sections and also reviewed and revised the manuscript. JP took part in the study planning and review meetings, helping to validate and select the inputs used to populate the model. JP also reviewed and revised the manuscript. All authors read and approved the final manuscript.

## Pre-publication history

The pre-publication history for this paper can be accessed here:

http://www.biomedcentral.com/1471-230X/14/137/prepub
